# Periodic Health Examination and Injury Prediction in Professional Football (Soccer): Theoretically, the Prognosis is Good

**DOI:** 10.1007/s40279-018-0928-y

**Published:** 2018-04-27

**Authors:** Tom Hughes, Jamie C. Sergeant, Danielle A. van der Windt, Richard Riley, Michael J. Callaghan

**Affiliations:** 1 Manchester United Football Club, AON Training Complex, Birch Road, Off Isherwood Road, Carrington, Manchester UK; 2 Arthritis Research UK Centre for Epidemiology, Centre for Musculoskeletal Research, Manchester Academic Health Science Centre, University of Manchester, Manchester, UK; 3 Centre for Biostatistics, University of Manchester, Manchester Academic Health Science Centre, Manchester, UK; 4 Centre for Prognosis Research, Research Institute for Primary Care and Health Sciences, Keele University, Staffordshire, UK; 5 Department of Health Professions, Manchester Metropolitan University, Brooks Building, Bonsall Street, Manchester, UK

## Abstract

In professional soccer and other elite sports, medical and performance screening of athletes (also termed periodic health examination or PHE) is common practice. The purposes of this are: (1) to assist in identifying prevalent conditions that may be a threat to safe participation, (2) to assist in setting benchmark targets for rehabilitation or performance purposes and (3) to assist clinicians in determining which athletes may be at risk of future injury and selecting appropriate injury prevention strategies to reduce the perceived risk. However, when using PHE as an injury prevention tool, are clinicians seeking to identify potential causes of injury or to predict future injury? This Current Opinion aims to examine the conceptual differences between aetiology and prediction of injury while relating these areas to the capabilities of PHE in practice. We also introduce the concept of prognosis—a broader approach that is closely related to prediction—and why this may have greater applicability to PHE of professional athletes.

## Key Points

**Table:** 

Periodic health examination (PHE) is commonly used in professional football and other elite sports to provide baseline physical measurements for rehabilitation or performance purposes and to assist in selection of injury prevention practices.
PHE is often used to identify possible contributing causal factors for injury. However, due to issues with analysis and confounding, this is unachievable.
Using PHE for injury risk prediction is theoretically achievable, but we suggest that using the related concept of prognosis is arguably more appropriate for professional athletes.

## Introduction

A 32-year-old professional football player is sprinting towards the goal. He feels sudden pain in his right hamstring, falls to the ground and cannot continue. Medical assessment reveals a torn right semimembranosus and he will miss the rest of the season. The medical staff might ask themselves: “Could our screening processes have identified possible causal factors or maybe predicted this injury? Could we have prevented it?”

The phrase “an ounce of prevention is better than a pound of cure” is as relevant as never before. In elite professional team sports such as football, preventing an injury is big business. For every player missing through injury the cost to an elite football team is approximately €20,000 (US$24,000) per day [1]. Limiting time lost through injury has significant positive implications for team performance [2] with successful teams and individual players receiving vast commercial and financial rewards [2, 3].

In elite sport, a key component of injury prevention practice is medical and performance screening, also termed periodic health examination (PHE) [4]. In European professional football, 94% of teams conduct PHE which usually consists of medical, musculoskeletal examination and performance tests during pre-season and in-season periods [5]. As well as providing regular health surveillance, PHE has other potential benefits, such as identification of prevalent musculoskeletal or medical pathology that may prohibit safe participation or limit performance [6]. PHE can also provide baseline measures to which clinicians can refer and monitor progress through rehabilitation should a player become injured and which sports scientists can use as markers for training response. PHE has an integral role in development of injury prevention strategies [7, 8]. Traditionally PHE has been used to identify factors that are potentially related to mechanisms causing pathology and guide early management of these factors [8]. Additionally, PHE can be used as a predictive tool to identify and manage factors or performance impairments [4] that could be associated with increased injury risk [6], even if they do not always contribute to the cause of injuries.

Using PHE to find and fix a potential problem before it happens seems a simple and logical approach, but in reality it is fraught with complexity. As such, not all PHE programmes are beneficial to athletes who have been evaluated [4, 6], which could be due to several reasons. Because of the relatively small number of injuries that occur in elite sport populations compared to the general population, there is limited evidence to guide practitioners in selection of valid, reliable, sensitive and specific tests appropriate for elite athletes [6]. A related issue is that for tests included in PHE batteries, establishing thresholds to determine whether athletes are considered as high or low risk can be problematic [4]. Psychological factors may be associated with musculoskeletal injuries and could have an important role in injury prevention [9, 10], although current PHE guidelines do not provide specific recommendations in terms of psychological evaluation [6]. Importantly, confusion with terminology and blurring of the complicated theoretical concepts of injury aetiology (investigation of cause) and prediction (investigation of future outcomes) within the literature and at a practical clinical level also mean it is difficult for clinicians to fully appreciate the capabilities and limitations of PHE in injury prevention. Therefore, the aim of this Current Opinion article is to examine the conceptual differences between aetiology and prediction, whilst highlighting their relevance to PHE in professional football and other sports.

## Periodic Health Examination (PHE) as an Aetiological Screening Model—the Impossible Goal?

**Fig. 1:**
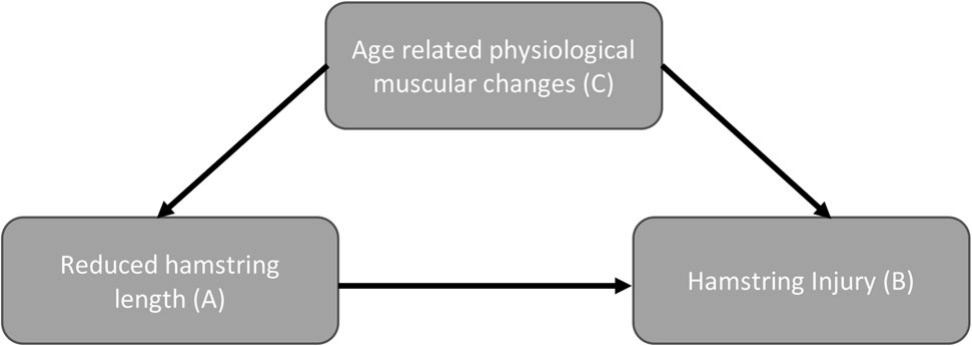
Diagram to show a simple causal pathway between hamstring length and hamstring injury, with age as a confounding factor

Aetiological research investigates mechanisms or factors that may cause injury, primarily using cohort studies [11, 12]. PHE is frequently perceived to be able to do the same: to identify likely causal factors that elevate the risk of future injury in a cohort of athletes. Clinicians may develop specific injury prevention strategies designed to modify such potentially causal factors [8] and therefore affect a reduction of the risk of a future injury [13, 14]. In our example (Fig. 1), suppose the player demonstrated reduced hamstring length (A) on the right leg during the muscle length testing component of PHE. It is tempting to assume that this was a cause of the hamstring injury (B). However, this interpretation is too simplistic.

A causal factor of an individual injury is any characteristic, variable or condition that must have been present before or at injury onset such that, if the frequency, value or quality of the factor was different in a particular way, the injury may not have occurred or it may have occurred after a latent period [15, 16]. With the exception of traumatic, impact-related injuries, individual causal factors are unlikely to cause an injury independently; most injuries are due to multiple factors [13]. Some factors may have a strong or weak influence on the risk of injury [17] and the relative influence of each is dynamic, often changing over time [13]. To confirm factors as causal (such as those investigated during PHE), a body of high quality evidence is required [12], but this is currently lacking for injuries specifically in professional football [18, 19] and is of variable quality in other sports [20–27]. Studies that investigate causal factors should provide detailed methodology and design, explicit definitions and measurement criteria for causal factors, confounding factors and pathological outcomes. These are outlined in reporting guidelines such as the Strengthening the Reporting of Observational Studies in Epidemiology (STROBE) statement [28].

A term frequently used in epidemiology is confounding. A confounding factor, put simply, is another variable that confuses our understanding of the relationship between what we think is a causal factor and the outcome. To be considered as a confounder, the variable must be associated with the injury outcome in its own right but also have an association with the supposed causal factor under investigation (e.g. observed in a PHE test) [29, 30]. In our example (Fig. 1), during PHE our player demonstrated reduced hamstring length. If it is assumed that reduced hamstring length (A) could be a causal factor for hamstring injury (B), age-related physiological muscular changes could be viewed as a confounding factor (C). A possible explanation is that age-related physiological changes could adversely affect hamstring length, but also may influence the risk of hamstring injury independently.

Other typical examples of confounders are ethnicity, height and gender. Confounding may lead to incorrect estimates of the association between a factor and injury and thereby distort our view of what may cause an injury if we do not appropriately account for them. Although not the focus of this article, confounding is usually controlled for in observational studies through study design methods (e.g. stratification and restriction) or multivariable statistical analysis (adjustment of the association) [12]. However, these methods cannot account for unknown or unmeasured confounding factors. Therefore, even in robust observational studies, it is nearly impossible to completely eliminate the influence of confounding factors [31].

It is acknowledged that in this Current Opinion, we have presented a simplification of causal factor and confounding relationships to injuries in order to maintain a clinical focus. However, these relationships usually have even greater complexity and, in particular, may include mediating factors (which may exist between a causal factor and outcome) and moderating factors (interactions between two factors). For detailed information about such complexities in terms of causation, readers are advised to refer to Corraini et al. [32] and Rothman, Greenland and Lash [16].

When PHE is conducted in practice, because of the basic descriptive statistical methods used, the absence of a strong evidence base to underpin PHE as a tool to assess possible causes of injury whilst not controlling for confounding factors, can we realistically expect PHE to inform clinicians about the possible causes of injury and therefore guide injury prevention strategies? We would suggest not.

## PHE as a Prediction/Prognostic Screening Model—the Possible Goal

Prediction research is the investigation of the probability or risk of future health outcomes over time using both clinical and non-clinical information [11, 33]. Similar to aetiological research, prediction research questions are usually addressed through cohort studies [11]. In sports medicine, this concept is also frequently perceived to be an aim of PHE when used for injury prevention; that is, evaluating asymptomatic athletes to establish whether some measurements may predict future injury [4]. The capabilities of PHE for injury prediction purposes have recently been questioned, because the continuous nature of data obtained from many tests mean it is difficult to determine appropriate cut-off points that categorise athletes into high or low-risk groups [4]. However, even though PHE may never be able to perfectly dichotomise or categorise athletes into those who will and those who will not experience injury, the information it gathers can still have predictive value [34].

Viewing professional, elite athletes as healthy or asymptomatic at the time of PHE may also have limited validity. They are not representative of the general population and place abnormal physical load on their bodies. The vast majority have experienced musculoskeletal injuries as a consequence of high-level training and competition exposure. As a result, multifactorial prevalent or transient chronic musculoskeletal disorders are common in sport [8], and may present in athletes at the time of PHE. Such disorders are frequently managed through medical means, exercise or training load modification, which allows high-level function with reduced or tolerable symptoms.

Therefore, a related and broader concept for sports medicine to consider is prognosis. Traditionally in medical language, ‘prognosis’ commonly refers to the expected course of an individual’s illness or injury, although this has been viewed as too general and has only limited clinical application [33]. Prognostic research, however, aims to understand and predict future outcomes in those with an existing condition or baseline health state (e.g. previous injury) that warrants medical or clinical evaluation [35]. This is applicable to PHE and means that at the time of assessment, if an athlete has had a previous injury or existing condition, or if being an elite athlete can indeed be considered a condition in itself, PHE can be used to investigate an athlete’s prognosis in terms of sustaining future injuries.

Prognosis research can suffer from methodological flaws, including insufficient reporting of methodology, participation rate and outcomes, for example, which have led to erroneous conclusions and significant consequences in medical practice [36]. In response, the PROGnosis RESearch Strategy (PROGRESS) Partnership has been formed, which is an international, interdisciplinary collaboration that aims to enhance the transparency, quality and impact of prognosis research [35]. PROGRESS have proposed a framework of four different types of prognosis research for people with a particular condition or starting point of interest: (1) summary of overall outcome risk; (2) identification of factors that are associated with (and therefore explain) changes in outcome risk across individuals; (3) development and validation of prognostic models that predict individual outcome risk conditional on multiple factors; and (4) the identification of factors and tests that predict individual response to a particular treatment (stratified medicine) [14, 35–38]. PROGRESS demonstrate the importance of improved prognosis research methods, in particular for large confirmatory studies to identify factors associated with health outcomes; more appropriate statistical analyses to develop and validate models for individual risk of future outcomes; and improved ways to utilise prognosis research information to impact upon individualised treatment or management strategies.

The PROGRESS framework is reflective of the aims of PHE when used in terms of injury prevention and can help to guide development of robust and high-quality PHE processes. When considering our example, the ‘summary’ component (overall prognosis) may quantify the proportion of footballers who develop hamstring muscle injuries by the end of a season and therefore provide information on clinically important outcomes. The ‘explain’ component relates to the identification of prognostic factors (measured through PHE) that are associated with hamstring injury risk across individuals. The ‘predict’ component relates to using several of these factors (also measured through PHE) in a prognostic model to predict hamstring injury risk for an individual and select specific management strategies to modify the risk.

Factors associated with injury and assessed through PHE have been given various names within the literature, which is also a source of confusion. The term ‘risk factor’ is often used but this can mean that such factors are causal [39, 40], whether this is intentional or not, and we would argue that it is more suited to the aetiological model. However, in those with existing conditions (such as elite football players), PROGRESS recommends using the term ‘prognostic factors’ (PFs), defined as any variable that is associated with (predictive of) clinical events (such as injury) in populations with a defined baseline state [14, 35]. We prefer the PROGRESS definition for outcomes in sports medicine, as it reflects that importantly, both causal and non-causal factors can be a PF, as long as they provide information that contributes toward outcome prediction [14]. Again, this is also more reflective of PHE in professional football, as there are no clear causal links between PHE assessment and injuries [18]. One of the objectives of prognosis research is to combine several PFs within a multivariable prognostic (or prediction) model to determine the absolute risk of an injury for an individual [14, 38]. These models can guide selection of tailored management strategies based on each individual risk estimate and PF profile [38]. This approach can translate to PHE in practice and is different to the traditional PHE injury prevention paradigm, where prevention strategies are selected based on whether an abnormal PHE finding could be a known or assumed causal factor for injury [8]. In contrast to aetiology, confounding becomes less problematic in prognosis, as potential confounders (if known) are prognostic factors in their own right, and can be included in a prognostic model if they sufficiently contribute to outcome prediction [14]. If we want to know the risk of an injury occurring, it is unimportant whether this is estimated using causal or non-causal factors and there is no necessity to unravel or eliminate these relationships [11, 12]. The key thing is that the developed prognostic (prediction) model is sufficiently accurate to help inform a player’s management.

This approach is appropriate to PHE in elite sport because any factor, test or measurement performed on players could be statistically analysed to estimate its value as a PF, both individually and in combination with other factors within a prognostic model. So, in our example (Fig. 1), a prognostic model might include both reduced hamstring length and age, as the confounding relationship is no longer important. As such, individual clubs could select bespoke PHE test batteries through either experiential or evidence-based means, and clinicians could evaluate whether any aspect of their selected PHE has prognostic value. This could improve time and service efficiency as PHE batteries could be streamlined to include only those tests that have confirmed prognostic value, as well as tests considered important for rehabilitation or performance purposes. Using a smaller selection of tests based upon a prognostic model may allow more regular PHE assessment and evaluation of changing levels of absolute risk over time. Identification of PFs could assist development of innovative PHE tests in future [37].

Despite these benefits, there are also challenges to consider when utilising prognostic models within sports medicine. The quality of data used to develop a model is critical to its performance [38, 41], so tests chosen for a club’s PHE process should be reliable and precise. Accurate injury outcome identification is imperative, using clinical criteria or gold standard diagnostic measures where possible, to reduce the effects of outcome misclassification [18, 42, 43]. Once developed, models should be validated internally, where predictive performance is tested on the dataset from which it was derived (e.g. using a resampling technique such as bootstrapping) with adjustments for overfitting/optimism made [44]. Ideally, validation should be completed externally on data from a sample from another location [38], but when considering our example, this would mean another football club. This, however, is unrealistic in the world of elite-level sports because achieving or maintaining a competitive edge is key, so all that matters in these situations is that predictive performance is acceptable and informative in the local setting.

Although an accurate prognostic model can assist clinicians in healthcare delivery, in order to be useful they should be easy to implement in clinical practice [38] and be directly relevant to the clinician’s available skillset and resources [45]. Using our example, if we could create a model that included hamstring length assessment as a PF for a structural hamstring injury, then clinicians would have consistent methods of hamstring length assessment, in addition to accurate and reliable diagnostic measures (such as imaging) to prevent misclassification. Importantly, although statistical models can be useful, they are a theoretical construct developed under scientific or mathematical assumptions whilst using real data. Although assumed to represent the real world, this may not necessarily be the case and so require a degree of cautiousness and pragmatism with interpretation [46]; prognostic models should help clinical decision making and not be seen as a replacement for this process [47].

## Conclusion and Recommendations

After the football player’s hamstring tear, the medical staff asked themselves: Firstly, could PHE have identified the cause of the injury? It is improbable that PHE would allow identification of the cause with certainty. Secondly, could they have predicted the injury risk using PHE? This is theoretically achievable, although currently the role of PHE in injury prognosis (or prediction) is unsubstantiated in professional football due to significant shortcomings in the quality and quantity of the current evidence [18]. Although further research is clearly essential to improve our understanding of this area, the discussed concepts based on our current knowledge have significant implications. If the aim of PHE is to set benchmarks for rehabilitation or performance targets, then the aetiology versus prognosis (prediction) debate is irrelevant. However, if PHE is to be used to inform injury prevention strategies, then we need to be explicit whether we are investigating cause or prognosis (prediction). We advocate applying the PROGRESS framework to PHE and using clear terminology. Instead of using terms such as risk factor, we should use prognostic factor for factors associated with outcomes, whether causal or non-causal, and reserve the term ‘causal factor’ for the rare occasions when we have the evidence to justify its use.

It is hoped that this Current Opinion may help dispel the myth that injury causality can be routinely established through PHE. Instead, using the fundamentals of prognosis research can allow a data-guided, bespoke estimation of risk and inform possible injury prevention rather than being led by limited evidence, unconfirmed hypotheses and clinical intuition alone.
